# Identification of Amino Acids within Nonstructural Proteins 10 and 14 of the Avian Coronavirus Infectious Bronchitis Virus That Result in Attenuation *In Vivo* and *In Ovo*

**DOI:** 10.1128/jvi.02059-21

**Published:** 2022-03-23

**Authors:** Sarah Keep, Phoebe Stevenson-Leggett, Giulia Dowgier, Holly Everest, Graham Freimanis, Michael Oade, John A. Hammond, Maria Armesto, Rut Vila, Tura Bru, Harm Geerligs, Paul Britton, Erica Bickerton

**Affiliations:** a The Pirbright Institute, Surrey, United Kingdom; b Zoetis, VMRD, Zaventem, Belgium; University of Kentucky College of Medicine

**Keywords:** coronavirus, infectious bronchitis virus, avian, vaccine, replicase, reverse genetics, avian viruses, reverse genetic analysis, veterinary vaccine development

## Abstract

The *Gammacoronavirus* infectious bronchitis virus (IBV) is a highly contagious global pathogen prevalent in all types of poultry flocks. IBV is responsible for economic losses and welfare issues in domestic poultry, resulting in a significant risk to food security. IBV vaccines are currently generated by serial passage of virulent IBV field isolates through embryonated hens’ eggs. The different patterns of genomic variation accumulated during this process means that the exact mechanism of attenuation is unknown and presents a risk of reversion to virulence. Additionally, the passaging process adapts the virus to replicate in chicken embryos, increasing embryo lethality. Vaccines produced in this manner are therefore unsuitable for *in ovo* application. We have developed a reverse genetics system, based on the pathogenic IBV strain M41, to identify genes which can be targeted for rational attenuation. During the development of this reverse genetics system, we identified four amino acids, located in nonstructural proteins (nsps) 10, 14, 15, and 16, which resulted in attenuation both *in vivo* and *in ovo*. Further investigation highlighted a role of amino acid changes, Pro85Leu in nsp 10 and Val393Leu in nsp 14, in the attenuated *in vivo* phenotype observed. This study provides evidence that mutations in nsps offer a promising mechanism for the development of rationally attenuated live vaccines against IBV, which have the potential for *in ovo* application.

**IMPORTANCE** The *Gammacoronavirus* infectious bronchitis virus (IBV) is the etiological agent of infectious bronchitis, an acute, highly contagious, economically important disease of poultry. Vaccination is achieved using a mixture of live attenuated vaccines for young chicks and inactivated vaccines as boosters for laying hens. Live attenuated vaccines are generated through serial passage in embryonated hens’ eggs, an empirical process which achieves attenuation but retains immunogenicity. However, these vaccines have a risk of reversion to virulence, and they are lethal to the embryo. In this study, we identified amino acids in the replicase gene which attenuated IBV strain M41, both *in vivo* and in *ovo*. Stability assays indicate that the attenuating amino acids are stable and unlikely to revert. The data in this study provide evidence that specific modifications in the replicase gene offer a promising direction for IBV live attenuated vaccine development, with the potential for *in ovo* application.

## INTRODUCTION

*Orthocoronavirinae* is a subfamily of the *Coronaviridae* family, in the order *Nidovirales*, and is divided into four genera: *Alphacoronavirus*, *Betacoronavirus*, *Gammacoronavirus*, and *Deltacoronavirus* (1). Coronaviruses belonging to each of these genera pose significant threats to human health, animal health, and animal welfare, as well as to food security. Notably, some betacoronaviruses are zoonotic, including severe acute respiratory syndrome coronavirus (SARS-CoV), which caused over 800 deaths from 2003 to 2004 (2, 3); Middle East respiratory syndrome coronavirus (MERS-CoV), first identified in 2012 (4); and SARS-CoV-2, which emerged in 2019, causing a worldwide pandemic (5). Other notable coronaviruses include the recently emerged porcine *Deltacoronavirus* (PDCoV), first reported in Hong Kong in 2012, which causes acute diarrhea, vomiting, dehydration, and mortality in piglets (6, 7), and recent isolates of the *Alphacoronavirus* porcine epidemic diarrhea virus (PEDV), which have caused mortality and economic losses to porcine industries in Europe and North America (8, 9).

The avian *Gammacoronavirus* infectious bronchitis virus (IBV) is the etiological agent of infectious bronchitis, a globally distributed, acute, highly infectious respiratory disease of domestic fowl. Birds infected with IBV via inhalation, direct contact with other infected birds, or contaminated fomites such as litter display a variety of clinical signs, including snicking, rales, nasal discharge, watery eyes, reduced weight gain, and lethargy. IBV primarily replicates in the epithelial cells of the upper respiratory tract but can also infect epithelial cells of the kidney, enteric tract, and oviducts (10–12). Infection can therefore lead to nephritis as well as decreases in egg production and quality. A major characteristic of IBV infection is a reduction of the ciliary activity of tracheal ciliated epithelial cells, commonly referred to as ciliostasis when ciliary activity is abolished (13). Ciliostasis often leads to mortality as infected birds become much more susceptible to secondary bacterial infections. Consequently, IBV infection and the resulting disease represent a major threat to the health and welfare of infected birds, and additionally result in a huge economic effect on global poultry production.

There are many IBV strains and variants which are categorized by both serotype and genotype (14). Several strains and variants can cocirculate and, although geographical regions often have specific ones of concern, some serotypes and genotypes are of global concern, including M41, a Massachusetts serotype, GI-1 genotype virus (14, 15). IBV is currently controlled using live attenuated vaccines, which are produced by the empirical method of serial passage of a virulent field isolate in embryonated hens’ eggs, typically more than 80 passages (16). A balance must be achieved between the accumulation of attenuating mutations and the retention of immunogenicity. The precise molecular mechanism of attenuation is unknown (17), and therefore different vaccines can have varying levels of efficacy. Vaccine viruses generated in this manner cannot be administered *in ovo* as the passaging process ultimately adapts the virus to growth in the embryo, subsequently increasing embryo lethality. Although vector vaccines and nucleic acid vaccines, such as those currently employed against SARS-CoV-2 (18–20), have been investigated, the efficacy of such vaccines was not considered high enough for use in poultry (21–27). Additionally, the administration route of such vaccines was not favorable for mass vaccination of poultry flocks (23, 24); IBV vaccines are often administered *en masse* as a spray or via drinking water (15). Consequently, a better understanding of IBV replication, and pathogenic determinants in particular, is required to aid in the design of live rationally attenuated vaccines.

IBV, like other members of the *Orthocoronavirinae* subfamily, has a large, positive-sense, single-stranded RNA genome, 27.6 kb, which possesses a 5′-terminal m7GpppN-cap and a 3′-terminal poly(A) tail. The 5′-proximal two-thirds of the genome comprises the replicase gene. This consists of two open reading frames (ORFs), designated 1a and 1ab, which are translated into two large polyproteins, pp1a and pp1ab, with the latter translated as the result of a -1 ribosomal frameshift mechanism (28, 29). Both polyproteins are proteolytically cleaved by virus-encoded proteases, resulting in the generation of 15 nonstructural proteins (nsps) that assemble to form replication-transcription complexes (RTC). Although the functions and roles of nsps in coronavirus replication are still being investigated, the functions of some are known, including nsp 12, an RNA-dependent RNA polymerase; nsp 13, an RNA helicase; nsp 14, an *S*-adenosyl methionine (SAM)-dependent (guanine-N7) methyl transferase (N7-MTase) and 3′–5′ exoribonuclease (ExoN); nsp 15, a manganese-dependent endoribonuclease (NendoU); and nsp 16, a 2′*O*-methyltransferase (30, 31). The 3′-proximal third of the IBV genome encodes the structural proteins spike (S), membrane (M), envelope (E), and nucleocapsid (N), as well as the accessory genes 3a, 3b, 5a, and 5b. Additional accessory genes, 4b and 4c, have been identified located between genes M and 5, corresponding to the previously designated intergenic region (32, 33). An ORF, 7, has also been identified, located between the N gene and the 3′ untranslated region (UTR) (33, 34).

Understanding of the proteins encoded by coronavirus genomes, including the IBV genome, and knowledge of viral replication and pathogenic and immunogenic determinants has been greatly enhanced through the development of multiple reverse genetic systems, which allow for targeted manipulation of the genome (35–40). Through reverse genetics, several pathogenic determinants within the IBV genome have been identified, including the accessory proteins 3a, 3b, 5a, and 5b (39, 41, 42), the spike gene (39), and the replicase gene (43). Rational manipulation of pathogenic and immunogenic factors within the IBV genome has been shown to offer a mechanism for the potential development of rationally attenuated vaccines (27, 42, 44, 45). This also applies to the wider field of coronavirus research, in which mutation of specific residues within nsps has been shown to result in attenuated *in vivo* phenotypes, including residues in the ExoN domain within nsp 14 (46, 47), residues within nsp 15 (48, 49), and residues of the conserved KDKE motif of nsp 16 (50, 51).

In this publication, we describe the successful development of a reverse genetic system for the pathogenic IBV M41-CK; a lab-adapted isolate of the M41 strain, a Massachusetts serotype virus that is of concern to poultry industries globally (15). Despite lab adaption to replication in primary chicken kidney (CK) cells, M41-CK has a pathogenic phenotype *in vivo* (44). In addition to developing a reverse genetics system based on a pathogenic IBV, we identified two amino acids located in ORF1ab, specifically in nsps 10 and 14, that were responsible for the attenuation of a recombinant M41 both *in vivo* and *in ovo*. The attenuating amino acids were identified from natural variant sequences of M41, and not from using *in silico* methods to identify potential amino acids involved in inactivating or modifying proteins encoded in the nsps. These findings further support those of our previous research, suggesting that the ORF1ab of IBV is a determinant of pathogenicity (43) and has a wider role in coronavirus pathogenicity, and that mutations within nsps 10 and 14 may result in attenuated phenotypes in human coronaviruses.

## RESULTS

### The generation of an infectious, full-length molecular clone of M41-CK.

**FIG 1:**
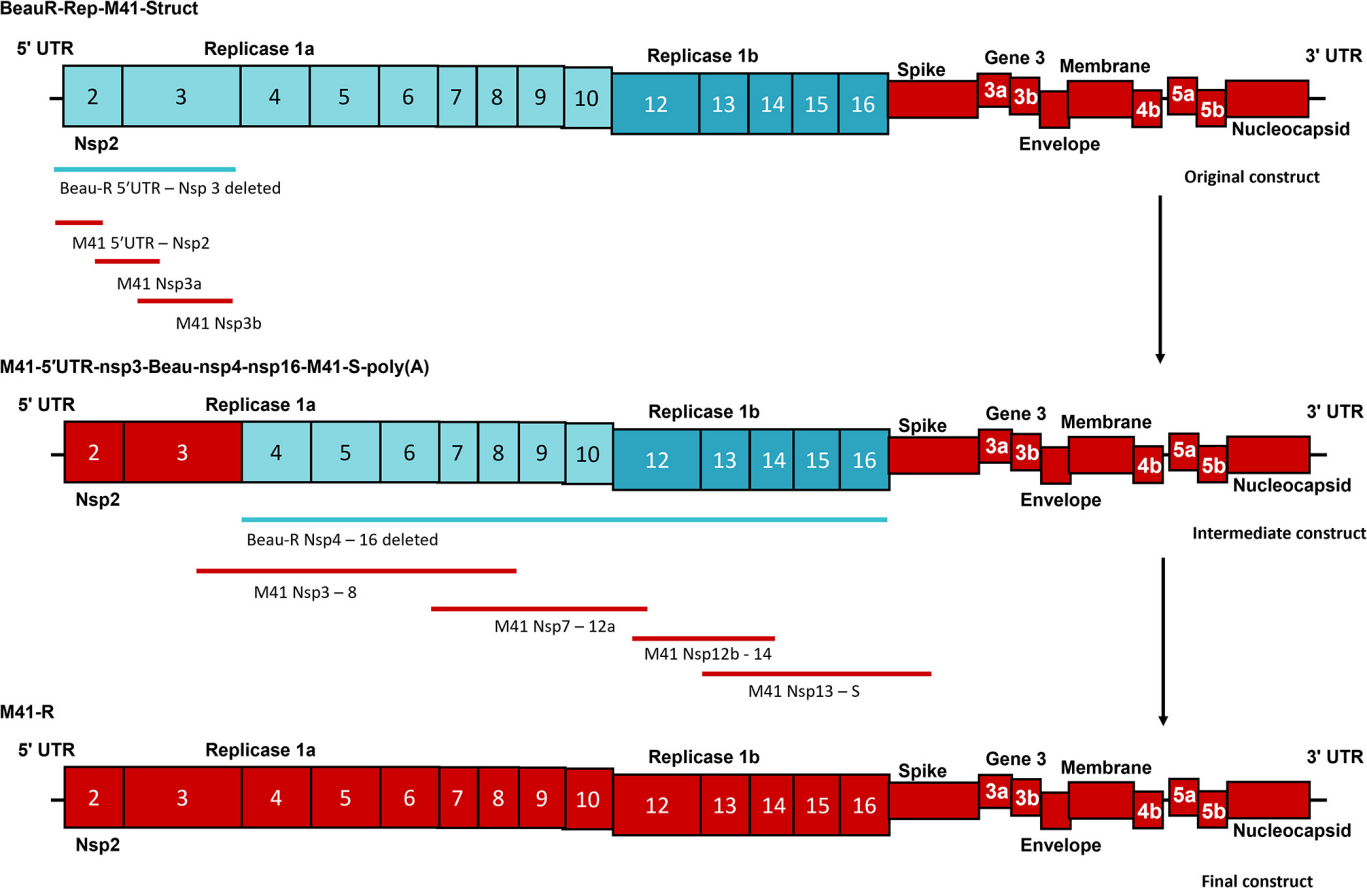
Schematic detailing the assembly of the cDNA copy of the M41-CK genome. The construction of the cDNA encoding the full-length copy of the M41-CK genome was a multi-step process which started with a recombinant vaccinia virus encoding the cDNA BeauR-Rep-M41-Struct (43). Using this hybrid IBV cDNA construct, the Beau-R sequence (blue) encoding 5′UTR nsp 2 and 3 was deleted and sequentially replaced, using two RT-PCR-generated and one chemically synthesized cDNA fragment, with the corresponding M41-CK sequence (red), generating an intermediate hybrid IBV cDNA denoted M41-5′UTR-nsp3-Beau-nsp4-16-M41-S-poly(A). At the immediate 5′ end of the IBV cDNA, this intermediate hybrid contained a T7 RNA promoter sequence for generating infectious RNA. The remaining Beau-R replicase sequence was deleted from the intermediate IBV cDNA construct and sequentially replaced, using four chemically synthesized fragments, with the corresponding M41-CK polymerase sequence, leading to the generation of a full-length cDNA copy of the M41-CK genome under the control of a T7 RNA promoter with a hepatitis delta ribozyme-T7 terminator sequence immediately adjacent to a 25-nucleotide poly(A) tail within the vaccinia virus genome.

A cDNA copy of the IBV M41-CK genome was assembled within a recombinant vaccinia virus (rVV) using a multi-step process which is summarized in Fig. 1. In summary, a rVV containing the IBV sequence BeauR-Rep-M41-Struct (43), in which the 5′ UTR and ORF1ab sequence consists of Beau-R sequence and the remaining sequence corresponds to the structural accessory genes and the 3′ UTR derived from M41-CK, was used as the starting point. A full-length genomic sequence of M41-CK within the thymidine kinase (TK) gene of the rVV genome was produced by deleting the remaining Beau-R sequence in rVV BeauR-Rep-M41-Struct (43), which was then sequentially replaced with the corresponding M41-CK sequence via homologous recombination using transient dominant selection (52). Two rVVs, rVV M41-R-6 and rVV M41-R-12, containing the full-length M41-CK sequence and generated through independent recombination events, were isolated and the M41-CK full-length inserts were sequenced. The M41-R cDNA sequences in both rVV M41-R-6 and rVV M41-R-12 were identical to each other and to our “in-house” reference M41-CK sequence which was generated from virus passaged in cell culture. DNA was produced from both rVVs and used to recover two recombinant IBVs (rIBVs), M41-R-6 and M41-R-12, using previously published protocols (53). We always generate two randomly selected rIBVs to decrease the likelihood that a recovered virus may encode a deleterious mutation.

### *In vitro* and *ex vivo* replication of rIBVs M41-R-6 and M41-R-12 is comparable to that of M41-CK.

**FIG 2:**
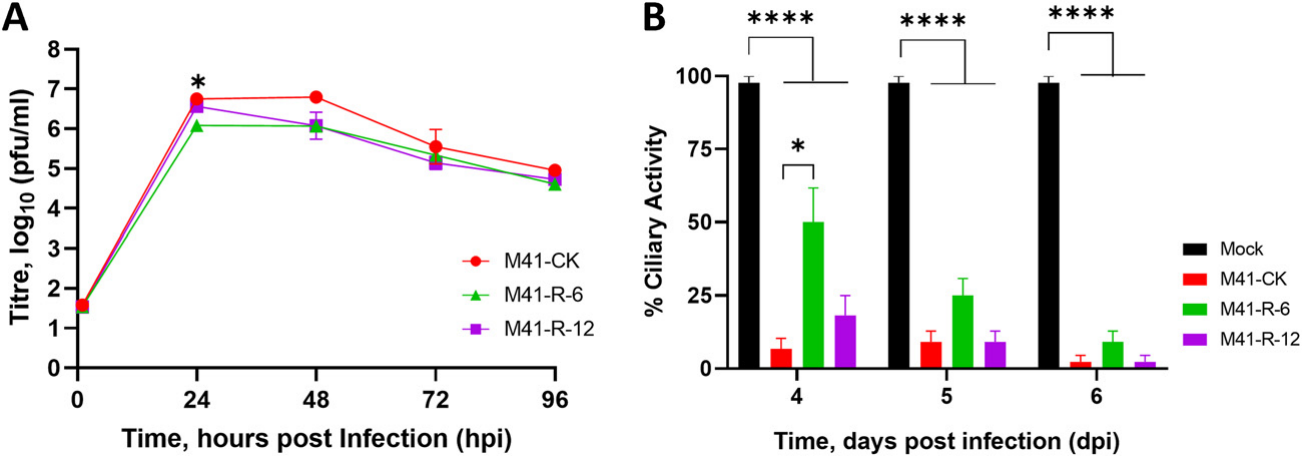
The replication kinetics of M41-R are comparable to those of M41-CK *in vitro* and in *ex vivo* TOCs. (A) Primary CK cells were inoculated with 10^5^ PFU of either rIBV M41-R-6, M41-R-12, or M41-CK. Supernatants were harvested at 24-h intervals and quantities of infectious progeny virus present were determined by plaque assay in CK cells. Each point represents the mean of three independent experiments with error bars representing standard error of the mean (SEM). (B) *Ex vivo* TOCs, prepared from 19-day-old SPF embryos, were inoculated in replicates of 11 with 10^4^ PFU of either rIBV M41-R-6, M41-R-12, M41-CK, or medium for mock infection. Ciliary activities were assessed by light microscopy at regular intervals and the mean activities of 11 replicates were calculated. Error bars represent SEM. (A and B) Statistical differences were analyzed using a two-way ANOVA with Tukey analysis for multiple comparisons and are indicated by * (*P* < 0.05) and **** (*P* < 0.0001). For clarity, in panel A, * denotes that rIBV M41-R-6 is lower than both rIBV M41-R-12 and M41-CK at 24 hpi.

Both rIBVs, M41-R-6 and M41-R-12, displayed replication kinetics comparable to each other and to the parental M41-CK in a primary cell culture over 96 h (Fig. 2A). Tracheal ciliary activity is a well-established marker of IBV replication (54) in *ex vivo* tracheal organ cultures (TOCs); replication of the rIBVs M41-R-6 and M41-R-12 resulted in greater than 75% cessation of ciliary activity, and replication of parental M41-CK resulted in complete cessation of ciliary activity (ciliostasis) (Fig. 2B). These results indicated that both rIBVs had very similar phenotypes to parental M41 CK *in vivo* and *ex vivo*.

### rIBVs M41-R-6 and M41-R-12 do not display a pathogenic phenotype *in vivo*.

**FIG 3:**
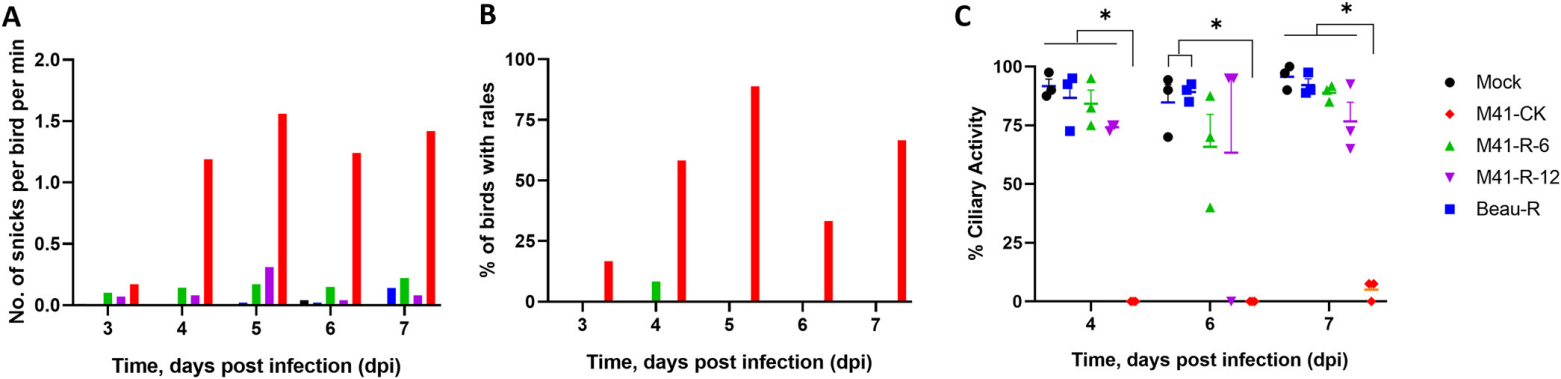
M41-R displays an attenuated phenotype *in vivo*. Groups of 12 8-day-old SPF RIR chicks were inoculated with 10^5^ PFU of Beau-R, rIBV M41-R-6, rIBV M41-R-12, or M41-CK via the intranasal and intraocular route. Mock-infected birds were inoculated with serum-free BES medium. Birds were observed for clinical signs from 3 to 7 dpi. (A) The average number of snicks per bird per minute and (B) the percentage of birds exhibiting rales per group. Only birds infected with M41-CK displayed these two clinical signs. (C) Ciliary activities were measured from tracheas harvested from three randomly selected birds per group at 4, 6, and 7 dpi. The average percentages for each group are displayed with error bars representing SEM. Statistical differences were analyzed using a two-way ANOVA with Tukey analysis for multiple comparisons. Statistical differences are indicated by * (*P* < 0.05). There were no statistical differences between the mock, the apathogenic control group Beau-R, M41-R-6, and M41-R-12 groups.

Groups of 8-day-old Rhode Island Red (RIR) specific-pathogen-free (SPF) chickens were infected, via the intranasal and intraocular routes, with 10^5^ PFU of the rIBVs Beau-R (38), M41-R-6 or M41-R-12, and IBV M41-CK, or mock-infected with serum-free medium. The rIBV Beau-R is attenuated *in vivo* (55) and was included as a known apathogenic control virus. The infected chickens were observed for IBV-associated clinical signs, snicking, rales (Fig. 3A and B), wheezing, nasal discharge, and watery eyes, at 24 h intervals from 3 to 7 days postinfection (dpi). Very few clinical signs were observed in the rIBV Beau-R-, M41-R-6-, and M41-R-12-infected groups in comparison to the M41-CK-infected group, which exhibited snicking from 4 to 7 dpi and rales from 3 to 7 dpi with a peak at 5 dpi of almost 90% (Fig. 3A and B).

Tracheal ciliary activity of epithelial cells is used as an *in vivo* marker for IBV pathogenicity, with a ciliary activity score of 0% indicating replicating, pathogenic IBV and an activity score of 100% indicating that no replicating IBV is present (54). Ciliary activities in the tracheas were assessed at 4, 6, and 7 dpi from three randomly selected chickens per group (Fig. 3C). The average ciliary activities observed from the rIBV M41-R-6- and M41-R-12-infected birds on 4, 6, and 7 dpi were similar to each other and, more importantly, were similar to those from the apathogenic rIBV Beau-R group and the mock-infected birds rather than those from the pathogenic M41-CK group (Fig. 3C).

No IBV-derived RNA was detected in tracheal epithelial cells obtained from the rIBV M41-R-6- and M41-R-12-infected birds on either 4 or 6 dpi, although viral RNA was detected from one bird infected with each rIBV at 7 dpi (Table 1). Infectious IBV was only re-isolated from a trachea sample from one bird infected with M41-R-12 at 4 dpi. In contrast, IBV-derived RNA was detected in the tracheas of most of the M41-CK-infected birds, and infectious IBV was recovered from all extracted tracheas in the M41-CK-infected birds. The lack of infectious virus recovered from tracheas of the rIBV M41-R-6- and M41-R-12-infected birds, the retention of ciliary activity, and the lack of IBV-associated clinical signs in comparison to the pathogenic M41-CK control group, demonstrated that both M41-R-6 and M41-R-12 displayed an attenuated phenotype *in vivo*.

**TABLE 1:** Number of birds in each group positive for the presence of IBV RNA and infectious virus^*a*^

Group	IBV-derived RNA			Infectious IBV	
	4 dpi	6 dpi	7 dpi	4 dpi	6 dpi
Mock	0/3	0/3	0/3	0/3	0/3
Beau-R	0/3	0/3	0/3	0/3	0/3
M41-R-6	0/3	0/3	1/3	0/3	0/3
M41-R-12	0/3	0/3	1/3	1/3	0/3
M41-CK	3/3	2/3	2/3	3/3	3/3

a Virus presence was determined from randomly selected birds; results are displayed as the number of positive birds/total number of birds sampled per group.

### Identification of potential nucleotides responsible for the attenuation of rIBVs M41-R-6 and M41-R-12.

The cDNAs used to generate rIBVs M41-R-6 and M41-R-12 were generated from our “in-house” M41-CK sequence, obtained by sequencing M41-CK isolated from CK cells. As a consequence of the unexpected attenuated *in vivo* phenotype of rIBVs M41-R-6 and M41-R-12, we compared our original “in-house” sequence to a more recent M41-CK sequence, derived by 454 sequence analysis of a laboratory stock of IBV M41-CK propagated in embryonated hens’ eggs, with a known pathogenic *in vivo* phenotype (GenBank accession number MK728875.1) (17). The latter sequence was produced after the assembly of the M41-R cDNA. Comparison of these sequences revealed that our original “in-house” M41-CK sequence, on which the cloning strategy was based, differed at several positions, with four single nonsynonymous differences in ORF1ab (Table 2); this is a region we have previously indicated as involved in loss of pathogenicity (43).

**TABLE 2:** Location of nucleotide differences between primary CK cell and egg-derived M41 CK sequences

Genome region	Nucleotide position^*a*^	M41-CK (MK728875.1)	Amino acid	M41-CK^*b*^	M41-R^*b*^	Amino acid
Nsp 10	12,137	C	Pro	U	U	Leu
Nsp 14	18,114	G	Val	C	C	Leu
Nsp 15	19,047	U	Leu	A	A	Ile
Nsp 16	20,139	G	Val	A	A	Ile

a All nucleotide positions are based on the AY851295.1 sequence.

b Full-genome sequences based on our M41 CK cell-derived sequence.

**FIG 4:**
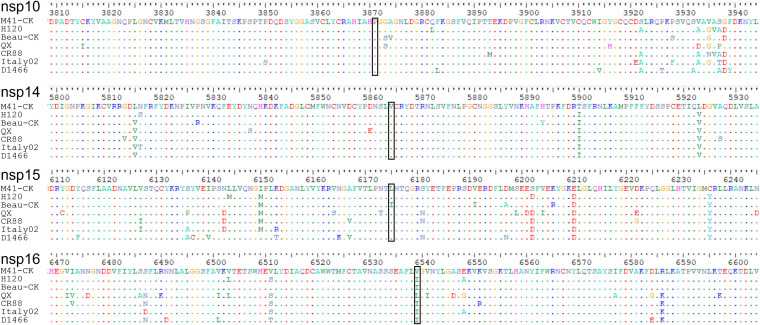
Proline85 in nsp 10 and valine393 in nsp 14 are conserved among IBV strains. Genome alignment of the full consensus-level genome sequences, downloaded from GenBank, of the following IBV strains with known *in vivo* phenotypes: the vaccine strains H120 (MN548287) and CR88 (MN548285), the attenuated strain Beau-CK (AJ311317), and the pathogenic strains QX (MN548289), Italy-02 (MN548288), M41-CK (MK728875.1), and D1466 (MN548286). Proline85 in nsp 10 and leucine393 in nsp 14 appear to be conserved among the IBV strains. Black box indicates the location of the substituted residues identified.

We hypothesized that the four amino acid differences, within nsps 10, 14, 15, and 16, observed between the original CK cell-derived M41-CK sequence and the sequence from virus grown in embryonated eggs (Table 2) may have a role in attenuation. To support this hypothesis, the full genome sequences of a variety of IBV strains with various well-characterized *in vivo* phenotypes were analyzed (Fig. 4). These included the IBV vaccine strains H120 and CR88, the attenuated laboratory strain IBV Beau-CK, and the pathogenic IBV strains D1466, Italy-02, and QX (34). Proline85 and valine393 in nsps 10 and 14, respectively, were found to be conserved among all the strains investigated. Leucine 183 within nsp 15 was found to be conserved in all but the attenuated Beau-CK strain, which has an isoleucine; the same as M41-R. Valine209 in nsp 16 showed the most variety, with QX containing a leucine and the remainder an isoleucine.

Previous research hypothesized that avirulent viruses existed as a minority species within a virulent population (17). Unfortunately, it was not possible to investigate the *in vivo* phenotype of the original stock of M41-CK passaged in CK cells, from which the historical sequence was derived. We analyzed virus populations from a more recently embryonated egg-passaged M41-CK with a known pathogenic *in vivo* phenotype. The viruses were deep sequenced to ultra-high depth using Illumina HiSeq. Two independent variant callers were used to identify single nucleotide polymorphisms (SNPs) present, focusing on four nucleotide positions: 12,137, 18,114, 19,047, and 20,139. The first variant caller, SiNPle (56), identified all four of the variations C12137U, G18114C, U19047A, and G20139A within the population, albeit at low frequencies of <1%: 0.02%, 0.01%, 0.15% and 0.11%, respectively. Using the second variant caller, LoFreq* (57), only the nucleotide changes U19047A and G20139A were identified, at rates of 0.15% and 0.12%, respectively, providing evidence that the nucleotides identified in our CK cell-passaged M41-CK are naturally present within IBV variants in a virus population that has an *in vivo* pathogenic phenotype. This presents circumstantial evidence that the M41-R sequence represents natural variant nucleotides within nsps 10, 14, 15, and 16, which may be responsible for the attenuated *in vivo* phenotype.

### Evaluation of the role of nsps 10, 14, 15, and 16 in IBV pathogenesis and the generation of a pathogenic rIBV.

To test our hypothesis that the four nucleotides in M41-R were responsible for attenuation, we produced a series of rVVs in which the M41-R-12 cDNA was modified to contain different combinations of the four nucleotides to match those identified in the pathogenic M41-CK egg-derived virus (Table 3). Due to the recombination-dependent nature of the vaccinia virus-based reverse genetics system, it was not feasible to change the distally located nucleotides in one recombination event. As such, two recombination events were used to replace the nucleotide residues in nsps 10, 14, 15, and 16 within the M41-R cDNA.

**TABLE 3:** Recombinant IBVs based on M41-R containing modified nucleotides in nsps 10, 14, 15, and 16^*a*^

IBV	Nsp				Amino acid changes
	10	14	15	16	
rIBV M41-R	U (Leu)	C (Leu)	A (Ile)	A (Ile)	
rIBV M41-K	**C (Pro)**	**G (Val)**	**U (Leu)**	**G (Val)**	Nsp10_L85P_-Nsp14_L393V_-Nsp15_I183L_-Nsp16_I209V_
M41-CK	**C (Pro)**	**G (Val)**	**U (Leu)**	**G (Val)**	
M41R-nsp10rep	**C (Pro)**	C (Leu)	A (Ile)	A (Ile)	Nsp10_L85P_
M41R-nsp10.14rep	**C (Pro)**	**G (Val)**	A (Ile)	A (Ile)	Nsp10_L85P_-Nsp14_L393V_
M41R-nsp10.15rep	**C (Pro)**	C (Leu)	**U (Leu)**	A(Ile)	Nsp10_L85P_-Nsp15_I183L_
M41R-nsp10.16rep	**C (Pro)**	C (Leu)	A (Ile)	**G (Val)**	Nsp10_L85P_-Nsp16_I209V_
M41R-nsp10.14.15rep	**C (Pro)**	**G (Val)**	**U (Leu)**	A (Ile)	Nsp10_L85P_-Nsp14_L393V_-Nsp15_I183L_
M41R-nsp10.14.16rep	**C (Pro)**	**G (Val)**	A (Ile)	**G (Val)**	Nsp10_L85P_-Nsp14_L393V_-Nsp16_I209V_
M41R-nsp10.15.16rep	**C (Pro)**	C (Leu)	**U (Leu)**	**G (Val)**	Nsp10_L85P_-Nsp15_I183L_-Nsp16_I209V_
M41R-nsp14.15.16rep	U (Leu)	**G (Val)**	**U (Leu)**	**G (Val)**	Nsp14_L393V_-Nsp15_I183L_-Nsp16_I209V_

a Boldface type indicates nucleotides derived from the pathogenic M41 CK sequence (GenBank accession no. MK728875.1, underlining represents nucleotides present in the apathogenic M41-R sequence). “rep” in the name of the rIBV stands for replaced and indicates the nucleotide replaced in the M41-R backbone. M41-K represents an rIBV, M41R-nsp10.14.15.16rep, in which all four of the M41-R nucleotides are replaced.

The first recombination event changed the nucleotide 12,137 in nsp 10 from U to a C, generating rVV M41R-nsp10replaced (M41R-nsp10rep). An rIBV, M41R-nsp10rep, was successfully generated from rVV M41R-nsp10rep (Table 3). Due to the close proximity of nsps 14, 15, and 16, a second recombination event was used to produce a series of modified M41-R cDNAs: initially, we used rVV M41-R, in which the M41-R version of nsp 10 was retained, and rVV M41R-nsp10rep, which resulted in either modification of nsps 14, 15, and 16, or in different combinations of the modified nsps, but all with nsp 10 corresponding to the pathogenic egg-derived M41-CK sequence. All the rVVs were used to successfully recover rIBVs containing each combination of the nucleotide residues, and sequence analysis confirmed that the rIBVs contained the replaced nucleotides (Table 3).

**FIG 5:**
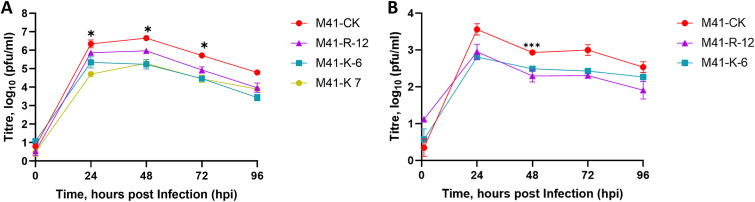
Incorporation of U12137C, C18114G, A19047U, and A20139G in nsps 10, 14, 15, and 16, respectively, within rVV M41-R generated a new rIBV, M41-K, which exhibits comparable replication kinetics *in vitro*. (A) Primary CK cells were inoculated with 10^5^ PFU of either rIBV M41-R-6, M41-R-12, or M41-CK. (B) *Ex vivo* TOCs, prepared from 2- to 3-week-old SPF chickens, were inoculated with 10^4^ PFU of either rIBV M41-R-12, M41-K-6, or M41-CK. Supernatants were harvested at 24-h intervals and the quantities of infectious progeny virus present were determined by plaque assay in CK cells. Each point represents the mean of three independent experiments with error bars representing SEM. Statistical differences were analyzed using a two-way ANOVA with Tukey analysis for multiple comparisons and are represented by * (*P* < 0.05) and *** (*P* < 0.0005). For clarity, the statistical difference highlighted in panel A denotes that the titers of both rIBV M41-K-6 and rIBV M41-K-7 are lower than that of M41-CK.

To determine whether the four nucleotides in the M41-R nsps 10, 14, 15, and 16 were responsible for the attenuated *in vivo* phenotype, the rIBV M41-K (Table 3), in which all four M41-R nucleotides were replaced with those identified in the pathogenic egg-passaged M41-CK sequence, was investigated both *in vitro* and *in vivo.* Two independent isolates of rIBV M41-K, rIBV M41-K-6 and rIBV M41-K-7, were rescued from two isolates of the rVV-M41-K. Growth kinetics in primary CK cells (Fig. 5A) demonstrated that both rIBV M41-K-6 and rIBV M41-K-7 displayed comparable replication kinetics to M41-CK and rIBV M41-R; therefore, rIBV M41-K-6 was used in subsequent experiments and referred to as rIBV M41-K. Growth kinetics in *ex vivo* TOCs (Fig. 5B) also showed that M41-K displayed comparable replication kinetics to rIBV M41-R and M41-CK.

**FIG 6:**
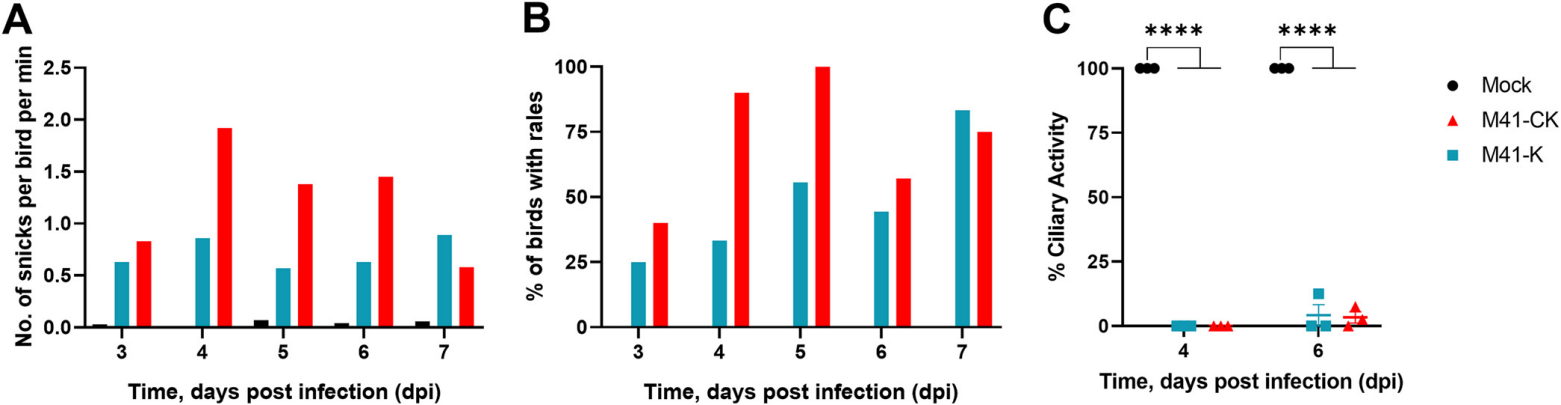
Incorporation of U12137C, C18114G, A19047U and A20139Fin nsp 10, 14, 15 and 16, respectively, within rVV M41-R generated a rIBV, M41-K, that exhibits a pathogenic phenotype *in vivo*. Groups of 12 8-day-old SPF RIR chicks were inoculated with 10^5^ PFU of rIBV M41-K or M41-CK via the intranasal and intraocular route. Mock-infected birds were inoculated with serum-free BES medium. Birds were observed for clinical signs from 3 to 7 dpi. (A) The average number of snicks per bird per minute and (B) the percentage of birds exhibiting rales per group were calculated. (C) Ciliary activities were measured from the tracheas harvested from three randomly selected birds per group on 4 and 6 dpi. Average percentages for each group are displayed with error bars representing SEM. Statistical differences were analyzed using a two-way ANOVA with Tukey analysis for multiple comparisons and are indicated by **** (*P* < 0.0001).

To determine the *in vivo* phenotype of rIBV M41-K, groups of 8-day-old SPF RIR chicks were infected with either rIBV M41-K or IBV M41-CK, or mock-infected (Fig. 6). Chickens infected with M41-K exhibited snicking and rales from 3 to 7 dpi, although the levels of clinical signs were lower than those of the M41-CK-infected group (Fig. 6A and B). The tracheal ciliary activities of chickens infected with rIBV M41-K or IBV M41-CK were comparable on both 4 and 6 dpi but significantly different from the ciliary activities observed in the tracheas of mock-infected birds (*P* < 0.0001) (Fig. 6C), indicating comparable replication of M41-CK and rIBV M41-K within tracheal ciliated epithelial cells. Reverse transcription-PCR (RT-PCR) analysis of tracheas extracted at 4 dpi identified IBV-derived RNA in two of three birds infected with rIBV M41-K or M41-CK (Table 4), and infectious IBV was recovered from all of the trachea samples. Viral RNA was detected in all of the tracheas, but infectious virus was only isolated from 1 of 6 or 2 of 4 birds infected with rIBV M41-K or M41-CK, respectively, at 7 dpi.

**TABLE 4:** Presence of IBV-derived RNA and infectious virus^*a*^

Group	IBV-derived RNA			Infectious IBV		
	4 dpi	6 dpi	7 dpi	4 dpi	6 dpi	7 dpi
Mock	0/3	0/3	0/6	0/3	0/3	1/6
rIBV M41-K	2/3	0/3	6/6	3/3	0/3	1/6
M41-CK	2/3	2/3	4/4	3/3	1/3	2/4

a Virus presence was determined from randomly selected birds; results are displayed as the number of positive birds/total number of birds sampled.

Our results established that replacement of the four nucleotides in nsps 10, 14, 15, and 16 within M41-R with those corresponding to the pathogenic egg-derived M41-CK sequence resulted in a new rIBV, M41-K, with a pathogenic *in vivo* phenotype. At least one of the four nucleotide changes was therefore responsible for the restoration of pathogenicity and, equally, at least one of the four nucleotides is responsible for the attenuated phenotype associated with M41-R. In addition, we have successfully produced a reverse genetics system for generating a pathogenic IBV.

### Identifying the minimal requirements for attenuation of IBV.

**FIG 7:**
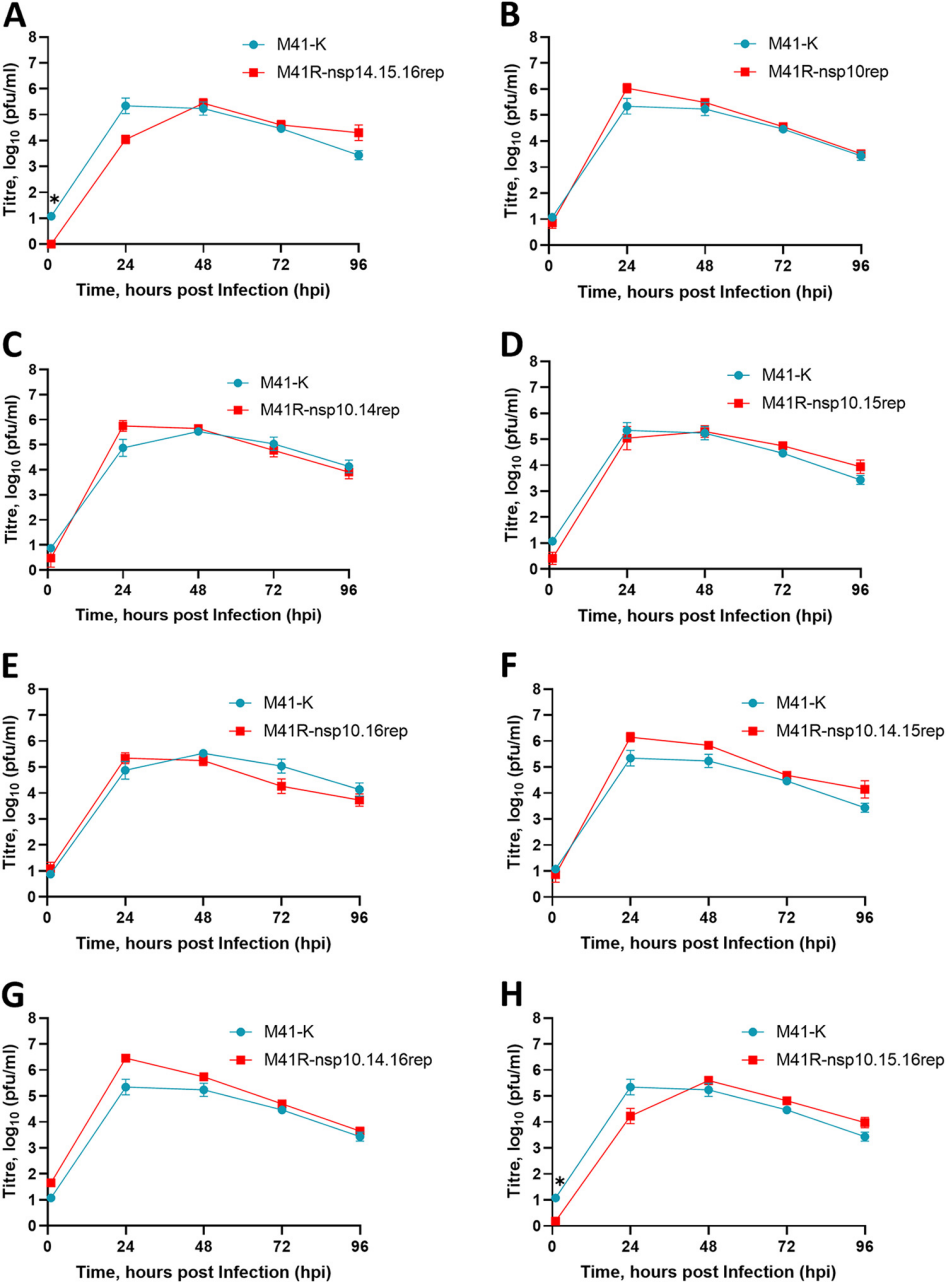
Combinations of the nucleotides identified at positions 12,137, 18,114, 19,047, and 20,139 in nsps 10, 14, 15, and 16, respectively, do not affect replication *in vitro*. A series of rIBVs were generated which contained either C/U, G/C, U/A, or G/A at positions 12,137, 18,114, 19,047, and 20,139 within nsps 10, 14, 15, and 16, respectively. All rIBVs were generated through modification of the M41-R backbone to replace (rep) the nucleotides at positions 12,137, 18,114, 19,047, and 20,139 to resemble the pathogenic M41-CK 454 sequence (GenBank accession no. MK728875.1). The IBVs are named according to the nsp location of the nucleotide which was replaced to resemble the latter sequence. Primary CK cells were inoculated with 10^4^ PFU of rIBV M41-K-6 and (A) rIBV M41R-nsp14.15.16rep (B) rIBV M41R-nsp10rep, (C) rIBV M41R-nsp10.14rep, (D) rIBV M41R-nsp10.15rep, (E) rIBV M41R-nsp10.16rep, (F) rIBV M41R-nsp10.14.15rep, (G) rIBV M41R-nsp10.14.16rep, or (H) rIBV M41R-nsp10.15.16rep. Supernatants were harvested at 24-h intervals and the quantities of infectious progeny virus present were determined by plaque assay in CK cells. Each point represents the mean of three independent experiments with error bars representing SEM. Statistical differences were assessed using a two-way ANOVA using Tukey analysis for multiple comparisons and are indicated by * (*P* < 0.05).

**FIG 8:**
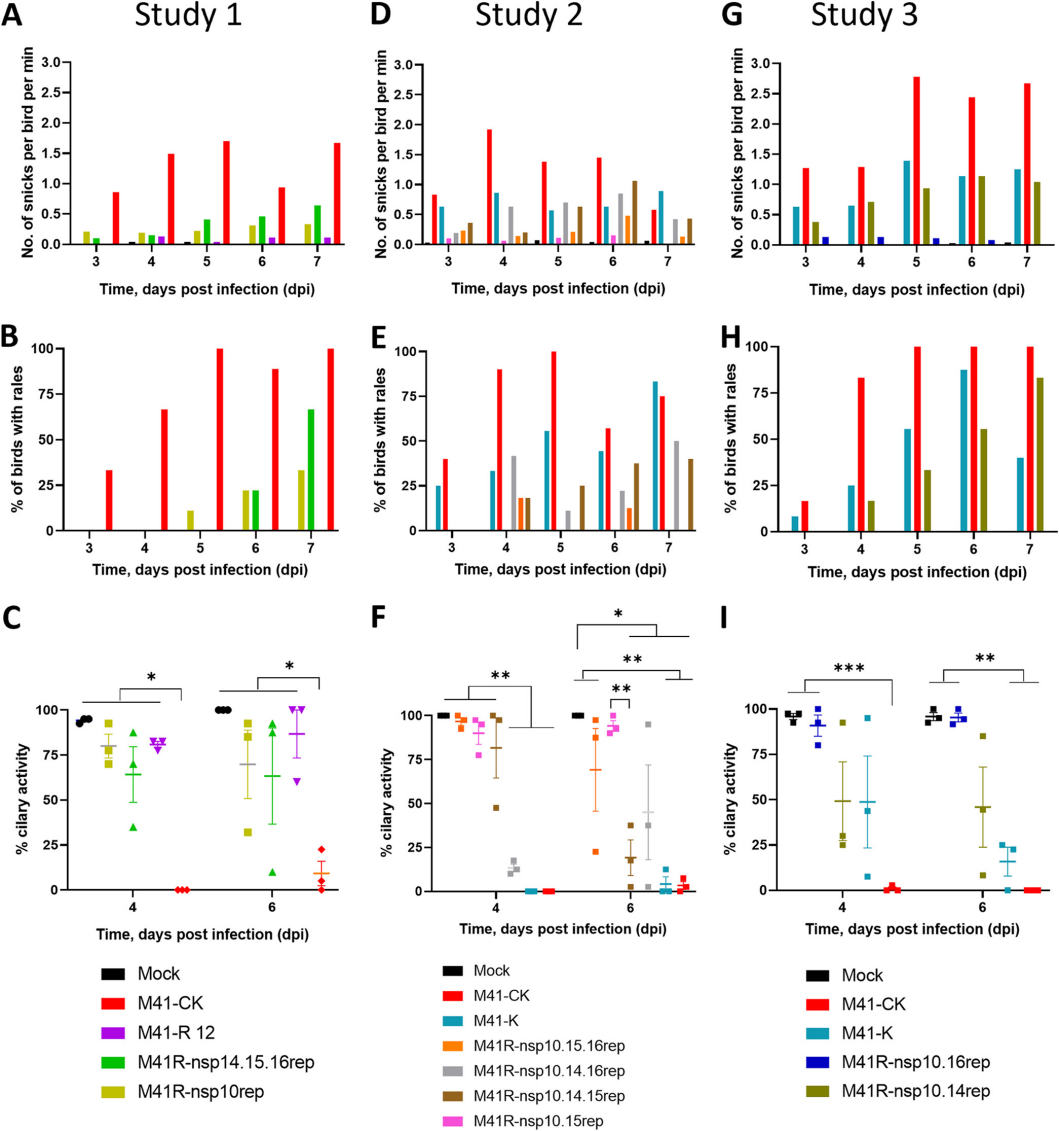
The attenuation of rIBV M41-R is the result of substitutions C12137U (Pro85Leu) in nsp 10, G18114C (Val393Leu) in nsp 14, and G20139A (Val209Ile) in nsp 16. A series of rIBVs were generated which contained either C/U, G/C, U/A, or G/A at positions 12,137, 18,114, 19,047, and 20,139 within nsps 10, 14, 15, and 16, respectively. All rIBVs were generated through modification of the M41 cDNA in rVV M41-R to replace (rep) the nucleotides at positions 12,137, 18,114, 19,047, and 20,139 to resemble the pathogenic egg-derived M41-CK sequence (GenBank accession number MK728875.1). Eight-day-old SPF RIR chicks were inoculated with 10^5^ PFU of IBV. Each experiment included a pathogenic M41-CK-infected group as well as a mock-infected group. The rIBVs assessed in each experiment are as follows: experiment 1 (A through C), rIBV M41-R, rIBV M41R-nsp14.15.16rep, and rIBV M41R-nsp10rep; experiment 2 (D through F), rIBV M41-K, rIBV M41R-nsp10.15.16rep, rIBV M41R-nsp10.14.16rep, rIBV M41R-nsp10.14.15rep, and rIBV M41R-nsp10.15rep; experiment 3 (G through I), rIBV M41-K, rIBV M41R-nsp10.16rep, and rIBV M41R-nsp10.14rep. In each experiment, birds were observed for clinical signs from 3 to 7 dpi. (A, D, G) The average number of snicks per bird per minute and (B, E, H) the percentage of birds exhibiting rales per group were calculated. (C, F, I) Ciliary activities were measured from tracheas harvested from three randomly selected birds per group at 4 and 6 dpi. The average percentage for each group is displayed with error bars representing SEM. Statistical differences were analyzed using a two-way ANOVA with Tukey analysis for multiple comparisons. Statistical differences are indicated by * (*P* < 0.05), ** (*P* < 0.005), and *** (*P* < 0.0005).

To determine which nucleotide or combination of nucleotide substitutions were associated with attenuation of IBV M41-CK, we produced a series of rIBVs which had various combinations of replaced nucleotides (Table 3). The *in vitro* growth kinetics of all rIBVs in primary CK cells were observed to be comparable to those of M41-K (Fig. 7). The pathogenicity of the rIBVs were tested in three separate *in vivo* studies alongside the pathogenic control viruses M41-CK and/or M41-K (Fig. 8). Mock-infected control groups were included in each experiment.

In study 1, we investigated the pathogenicity of the rIBVs M41R-nsp10rep and M41R-nsp14.15.16rep to determine whether nsp 10 versus nsps 14, 15 and 16 are involved in pathogenicity (Fig. 8A, B, and C). Chicks infected with rIBV M41R-nsp10rep or M41R-nsp14.15.16rep exhibited more observable clinical signs than those infected with rIBV M41-R, however, the levels of snicking and rales were lower than those of the M41-CK-infected group (Fig. 8A and B). Birds infected with M41R-nsp10rep and M41R-nsp14.15.16rep did exhibit rales at 6 and 7 dpi but, in contrast to those infected with M41-CK, did not exhibit rales at 3 or 4 dpi (Fig. 8B). Ciliary activities in the rIBV M41R-nsp10rep- or M41R-nsp14.15.16rep-infected chicks, although reduced on both 4 and 6 dpi, were not significantly different from the ciliary activities in the mock-infected or M41-R infected birds (Fig. 8C). The ciliary activities of M41-CK-infected chicks were significantly lower than those of all other groups on all days sampled (*P* < 0.05). The results for IBV-derived RNA and virus isolation from birds in the study groups are summarized in Table 5.

**TABLE 5:** Presence of IBV-derived RNA and infectious virus^*a*^

Expt.	Group	IBV-derived RNA			Infectious IBV		
		4 dpi	6 dpi	7 dpi	4 dpi	6 dpi	7 dpi
1	Mock	0/3	0/3	0/6	0/3	0/3	0/6
	M41-R	0/3	0/3	0/6	0/3	0/3	0/6
	M41R-nsp10rep	1/3	0/3	4/6	0/3	0/3	2/6
	M41R-nsp14.15.16rep	1/3	0/3	1/6	1/3	0/3	2/6
	M41-CK	2/3	1/3	2/6	3/3	1/3	1/6
2	Mock	0/3	0/3	0/6	0/3	0/3	1/6
	M41R-nsp10.15.16rep	0/3	1/3	2/5	2/3	0/3	1/5
	M41R-nsp10.14.16rep	3/3	1/3	3/6	3/3	2/3	0/6
	M41R-nsp10.14.15rep	3/3	3/3	2/5	2/3	3/3	0/5
	M41R-nsp10.15rep	0/3	0/3	0/6	0/3	0/3	0/6
	M41-K	2/3	0/3	6/6	3/3	0/3	1/6
	M41-CK	2/3	2/3	4/4	3/3	1/3	2/4
3	Mock	0/3	0/3	0/6	0/3	0/3	0/6
	M41R-nsp10.16rep	3/3	0/3	2/6	3/3	3/3	3/6
	M41R-nsp10.14rep	2/3	1/3	3/6	3/3	3/3	3/6
	M41-K	3/3	1/3	4/5	3/3	3/3	4/5
	M41-CK	3/3	1/3	1/6	3/3	3/3	4/6

a Virus presence was determined from randomly selected birds; results are displayed as the number of positive birds/total number of birds sampled. All positive RNA/virus isolation samples were spot-sequenced across nsps 10, 14, 15, and 16 to confirm the nucleotides at positions 12,137, 18,114, 19,047, and 20,139, respectively; no sequence changes were identified.

The ciliary activities demonstrated that the rIBVs M41R-nsp10rep and M41R-nsp14.15.16rep were apathogenic by the criterion of ciliary activity, although the presence of some clinical signs and the presence of virus in trachea samples from some chicks would suggest that they were not apathogenic to the same extent as observed for M41-R. In conclusion, replacing the M41-R nucleotide in nsp 10 alone or changing the nucleotides in nsps 14, 15, and 16 retained the attenuated phenotype. This indicated that the change in nsp 10 as well as either one, all or a combination of the changes in nsps 14, 15, and 16 within M41-R are required to restore the pathogenic phenotype of IBV M41-CK.

In study 2, we investigated the pathogenicity of four rIBVs, all containing the replacement of the nucleotide in nsp 10 of M41-R with replacement of combinations of nucleotides in nsps 14, 15, and 16. The rIBVs investigated were M41R-nsp10.14.15rep, M41R-nsp10.14.16rep, M41R-nsp10.15.16rep, and M41R-nsp10.15rep (Table 3). Both M41R-nsp10.14.16rep- and M41R-10.14.15rep-infected groups displayed clinical signs, including snicking and rales, from 4 to 7 dpi, similar to the rIBV M41-K-infected group (Fig. 8D and E). Ciliary activities were reduced in the rIBV M41R-nsp10.14.16rep-infected group on 4 and 6 dpi, and on 6 dpi for the rIBV M41R-nsp10.14.15rep-infected group compared to the mock-infected control group (*P* < 0.05), and were similar to those exhibited by both the M41-CK- and M41-K-infected groups (Fig. 8F). In contrast, birds infected with rIBVs M41R-nsp10.15.16rep and M41R-nsp10.15rep displayed reduced clinical signs in comparison to both the M41-K- and M41-CK-infected groups (Fig. 8D and E). Birds infected with M41R-nsp10.15.16rep retained ciliary activity at 4 dpi, which was reduced in one of the three birds at 6 dpi (Fig. 8F). IBV-derived RNA and virus isolation from birds are summarized in Table 5.

In summary, our results indicate that replacement of the M41-R nucleotide (U12137C; Leu85Pro) in nsp 10, in combination with nucleotide (C18114G, Leu393Val) in nsp 14, successfully conferred a pathogenic phenotype to M41-R. Replacement of the M41-R nucleotide in nsp 15 did not appear to be involved in the establishment of pathogenicity.

In study 3, we assessed rIBVs M41R-nsp10.14rep and M41R-nsp10.16rep to determine whether nsp 16, in the absence of replacing the nucleotide in M41-R nsp 14, played any role in restoring pathogenicity, and to confirm whether replacing nucleotides in nsps 10 and 14 in combination restored pathogenicity. Chickens infected with rIBV M41R-10.16rep displayed no clinical signs (Fig. 8G and H) and no significant reduction in ciliary activity compared with mock-infected birds at 4 and 6 dpi, respectively (Fig. 8I). Interestingly, IBV-derived RNA as well as infectious virus could be recovered on all sampling days (Table 5), indicating that M41R-nsp10.16rep is not completely attenuated in terms of *in vivo* viral replication.

Chickens infected with rIBV M41R-nsp10.14rep exhibited snicking and rales at broadly similar levels to the rIBV M41-K-infected group from 3 to 7 dpi (Fig. 8G and H). Average tracheal ciliary activities were reduced to <50% on both 4 and 6 dpi (Fig. 8I). Ciliary activities of birds infected with M41R-nsp10.14rep were statistically comparable to those of M41-K-infected birds on both 4 and 6 dpi, and infectious virus could be recovered, as in both pathogenic control groups, M41-K and M41-CK (Fig. 8I and Table 5). There was a larger range of ciliary activities observed in the rIBV M41R-nsp10.14rep group at 6 dpi compared to the M41-K group.

Overall, our results confirmed that replacing the nucleotides in nsp 10 (U12137C; Leu85Pro) and nsp 14 (C18114G; Leu393Val) of M41-R is sufficient to confer a pathogenic *in vivo* phenotype to rIBV M41-R and, in turn, the M41-R amino acids are responsible for the apathogenic phenotype associated with rIBV M41-R.

### Analysis of the rIBV M41-R as a potential *in ovo* vaccine.

Our *in vivo* data showed that rIBV M41-R is highly attenuated in chickens, which raises the potential for this virus as a candidate for future vaccine development. Vaccination *in ovo* is considered highly advantageous to the poultry industry; however, IBV vaccines are traditionally attenuated by multiple passages in embryonated eggs and consequently show increased lethality to embryos, observed as a drastic reduction in hatchability (58, 59). Our previous work on the attenuated rIBV Beau-R (38) for vaccine development (27, 44, 59, 60) showed that *in ovo* vaccination of 18-day-old SPF embryos resulted in 60% to 70% hatchability depending on dose (59). This hatchability, although considered too low for widescale commercial use, raised the possibility of using rIBVs as potential candidates for *in ovo* vaccination. To determine whether rIBV M41-R is also attenuated *in ovo* and to assess its potential for use as an *in ovo* vaccine, a hatchability study was performed. Groups of 18-day-old embryonated eggs were inoculated with a 50% egg infective dose (EID_50_) of either 10^1^ or 10^4^ rIBV M41-R. The embryonated eggs were allowed to hatch and the number of live chickens at 1-day-old was evaluated. At the lower dose of 10^1^ EID_50_, 32/33 (97%) of the rIBV M41-R-inoculated eggs successfully hatched; this was comparable to the mock-infected eggs at 29/33 (88%). A similar pattern was observed at the higher dose of 10^4^ EID_50_ M41-R with 29/33 (88%) successfully hatching. The hatchability in groups infected with M41-CK was 25/33 (76%) at the lower dose of 10^1^ EID_50_ and 21/33 (64%) at the higher dose of 10^4^ EID_50_. These results demonstrated that rIBV M41-R had a successful hatchability score, indicating that the virus is also attenuated *in ovo*.

### The nucleotides present in rIBV M41-R nsps 10, 14, 15, and 16 are stable during serial passage *in vitro* and *ex vivo*.

One potential risk associated with live attenuated vaccines is the possibility of reversion to a pathogenic phenotype. To investigate whether the nucleotides in nsps 10, 14, 15, and 16 of rIBV M41-R were likely to change and potentially result in a more pathogenic variant, rIBV M41-R was serially passaged in triplicate in CK cells and in replicates of four in *ex vivo* TOCs. Both rIBVs M41-R-6 and M41-R-12 were serially passaged 10 times in the primary CK cells and in TOCs, and the resulting viruses were sequenced across nsps 9, 10, 14, 15, and 16. No sequence changes were identified in the nsps of the passaged viruses from any of the replicates in either primary CK cells or *ex vivo* TOCs. The nucleotides associated with rIBV M41-R which have resulted in the attenuated *in vivo* phenotype are therefore stable up to at least passage 10 *in vitro* and *ex vivo*, further highlighting the potential of rIBV M41-R as a vaccine candidate.

## DISCUSSION

The emergence of the pandemic SARS-CoV-2 has highlighted the continuing need to understand the pathogenic determinants within a coronavirus genome. The advent of reverse genetics systems for coronaviruses in the early 2000s has enabled the study of pathogenic and immunogenic factors. For IBV, several reverse genetics systems have been developed by several research groups, initially based on the apathogenic Beaudette strain (38) and more recently on the attenuated vaccine strains H52 (37) and H120 (36), as well as the nephropathogenic QX strain (39). This study describes the successful development of a reverse genetics system based on the globally economically relevant pathogenic M41 strain. M41 belongs to the Massachusetts serotype; vaccines of this serotype are the most frequently used in vaccination programs across the globe (33). The development of this reverse genetics system therefore provides a powerful molecular tool for future in-depth research into IBV pathogenicity and immunogenicity factors, as well as for vaccine development.

During the development of the M41-based reverse genetics system, we identified four nucleotide substitutions within nsps 10, 14, 15, and 16 which resulted in a rIBV, designated M41-R, that exhibited an attenuated *in vivo* phenotype compared to the pathogenic strain M41-CK. The location of these nucleotides within ORF1ab supports previous research into IBV (27, 35, 36), as well as research into other coronaviruses such as MHV and SARS-CoV, which suggest that ORF1ab is a pathogenic determinant (37–40). Furthermore, several publications have linked nsps 10, 14, 15, and 16 to pathogenicity in other coronavirus, including SARS-CoV, MHV, and more recently SARS-CoV-2 (48–50, 61–63).

The amino acid residues described in this study were not identified as potential target residues which may affect associated enzymatic activities and potentially result in attenuation. Rather, an “in-house” sequence of M41-CK, generated pre-2005 from virus propagated in primary CK cells, was used as the template for designing oligonucleotides for assembling the replicase (ORF1ab) region of the M41-CK cDNA. Following recovery of two independent rIBV isolates, M41-R-6 and -12, based on our “in-house” M41-CK sequence, we found that the M41-R viruses grew as well as M41-CK *in vitro* in primary CK cells and *ex vivo* in TOCs (Fig. 2), but that they were attenuated *in vivo* (Fig. 3). Comparison of our CK cell-derived “in-house” sequence of M41-CK with a sequence of M41-CK derived from virus grown in embryonated eggs and known to be pathogenic identified four nonsynonymous nucleotide differences at positions 12,137, 18,114, 19,047, and 20,139 in nsps 10, 14, 15, and 16, respectively (Table 2), within ORF1ab. Comparison of our egg-derived M41-CK sequence to other IBV sequences of known pathogenicity which were not available when we designed our M41-CK replicase-associated oligonucleotides also showed that we had introduced four nucleotide changes into rIBV M41-R, compared to M41-CK, and that these changes were potentially responsible for the attenuated phenotype associated with the M41-R viruses. Interestingly, Illumina HiSeq sequencing on our egg-derived pathogenic M41-CK identified the four nucleotides associated with the attenuated rIBV M41-R, indicating that the attenuating nucleotides were potentially present as a variant within the virus population. As a consequence, a naturally occurring attenuated virus may have been selected through passage in primary CK cells. Nevertheless, the serendipitous introduction of these four nucleotides proved very useful in identifying amino acids associated with attenuation.

Using our reverse genetics system, we were able to modify the M41-R genome by systematically replacing the M41-R nucleotides with those from our pathogenic egg-derived M41-CK sequence; first, we completely restored virulence by replacing all four nucleotides and generating a pathogenic rIBV, designated M41-K. Further work, replacing combinations of nucleotides showed that the nucleotides present in M41-R nsp 10 and nsp 14 were required for restoring pathogenicity and were therefore responsible for attenuation. We cannot rule out the possibility that the nucleotide in nsp 16 plays a minor role in attenuation linked to virus growth. Our results indicate that the nucleotide in M41-R nsp 15 is not involved in restoring pathogenicity and may not play any role in attenuation.

Although research on coronavirus nsps 10, 14, and 16 has been carried out and crystal structures produced, this research has predominantly centered on SARS-CoV, MERS-CoV, MHV, and more recently SARS-CoV-2 (46, 64–70). There has been minimal research into these nsps regarding IBV. Nsp 10 is a small 15-kDa protein with two zinc fingers which are coordinated by a number of Cys and His residues, and it has been shown to play a role in the activation of both nsp 14 and nsp 16 (46–49). This protein is considered to be a cofactor for nsps 14 and 16; mutagenesis studies using MHV and SARS-CoV have also shown that nsp 10 is essential for viral replication (50, 51). Nsp 14 is a 59-kDa bifunctional protein consisting of two domains, a 3′–5′ ExoN domain and an *S*-adenosyl methionine (SAM)-dependent (guanine-N7) methyl transferase domain (52); the rIBV M41-R G18114C (Val393Leu) substitution is located in the latter domain. The ExoN domain has been shown to “proof-read” coronavirus RNA (71) and, more recently, has been demonstrated to play a role in RNA recombination (72). Interactions of nsp 14 with nsp 10 have been shown to enhance ExoN activity but have a minimal effect on N7-MTase activity (51). Nsp 16 is a 2′*O*-methyltransferase involved in the capping of viral RNA alongside nsp 14 (73). Nsp 16-mediated methylation has been shown to be sequence-dependent, with methylation occurring on the exposed 2′OH at the N7 position of the guanyl cap (74). More recently, SARS-CoV-2 nsp 16 has been shown to disrupt global mRNA splicing, thereby hampering the host cell innate immune response (75).

**FIG 9:**
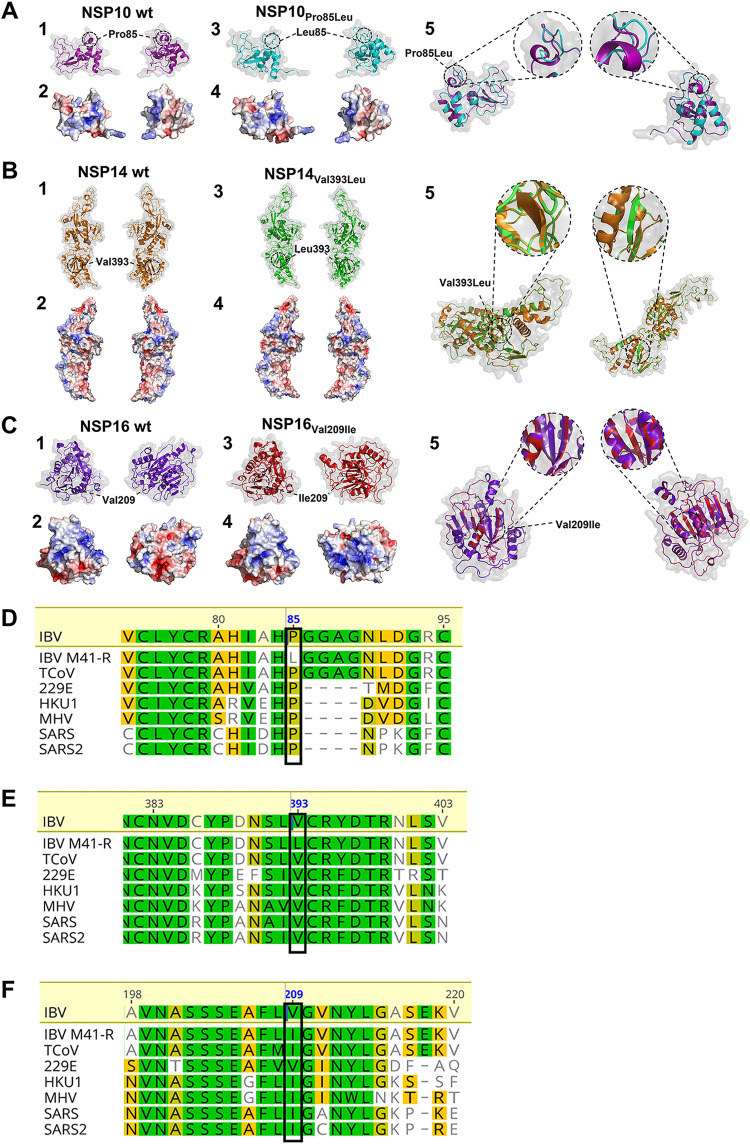
The amino acids at residue 85 in nsp 10 and 393 in nsp 14 are conserved among members of the coronavirus family with the change Pro85Leu in Nsp 10 predicted to affect the secondary protein structure. (A through C) Structural models of WT M41-CK nsp 10 and nsp 10 containing Pro85Leu, (E) WT M41-CK nsp 14 and nsp 14 containing Val393Leu, and (F) WT M41-CK nsp 16 and nsp 16 containing Val209Ile. All models were generated using SWISS-MODEL and visualized using PyMol using crystal structures of SARS-CoV nsps 10, 14, and 16 as the templates, RCSB PDB, 2XYV; RCSB PDB, 5C8T; and RCSB PDB, 2XYV; respectively. Each panel highlights specific viewpoints, with panels D2, D4, E2, E4, F2, and F4 highlighting the electrostatic mapping. Modeling of nsp 10 indicated that the presence of a leucine instead of a proline at residue 85 caused the loss of an α-helix and replaced it with an extended loop. The location and structural difference are highlighted in panel A5. (D to F) Amino acid alignment comparing the amino acid sequence in rIBV M41-R to HCoV-229E (GenBank accession no. KF514433), HCoV-HKU1 (NC_006577), IBV M41-CK (MK728875), MHV (KF268339), SARS-CoV (KF514395), SARS-CoV-2 (NC_045512), and turkey coronavirus (TCoV) (NC_010800). (D) Boxes indicate residue 85 in nsp 10, (E) residue 393 in nsp 14, and (F) residue 209 in nsp 16. MAFTT alignment has been generated using Geneious version 10.2.3 with colors displaying the percentage of similarity following Blosum80 score matrix.

Although several studies have identified several residues critical for the interactions of nsp 10 with nsps 14 and nsp 16 (64, 76, 77), and residues critical for the enzyme function of nsps 14 (78, 79) and 16 (74), the residues present in rIBV M41-R are not among those previously identified. This is not unexpected, as mutagenesis studies have identified residues that negatively impact viral replication; our research demonstrates that both *in vitro* and *ex vivo*, rIBV M41-R and all of the other rIBVs, including rIBV M41-K, are able to replicate comparably to the parental M41-CK strain *in vitro* (Fig. 2, 5, and 7). Structural modeling indicates that the Pro85Leu substitution in rIBV M41-R nsp 10 may impact the secondary structure of the protein, with the leucine residue resulting in the loss of an α-helix (Fig. 9A). No structural impact is predicted for the rIBV M41-R nsp 14 Val393Leu or the Val209Ile nsp 16 substitutions (Fig. 9B and C). Interestingly, the amino acid change in rIBV M41-R Nsp 14 falls within the N7-MTase domain, though it cannot have a significant effect on enzymatic activity since rIBV M41-R replicates as well as M41-CK in both primary CK cells and TOCs. However, it plays a major role in attenuation, indicating that the effect maybe host-based. A recent paper by Zhang et al. (2021) (63) modified the MHV nsp 14 by mutating the residue Y414A, having previously identified that Y414 is involved in the structure of the MHV N7-MTase, specifically located in the SAM binding site, and that it affects N7-MTase activity. The mutant virus was slightly attenuated in cell culture and significantly attenuated *in vivo*. The MHV Y414A mutation is 22 amino acid residues from the IBV Nsp 14 V393 residue, indicating that the M41-R V393L change may also affect N7-Mtase; however, we saw no attenuation in cell culture and restoration to virulence also required the change in nsp 10. However, this MHV study supports our conclusion that changes in the nucleotide sequence of IBV nsp 14 resulted in attenuation.

The exact mechanism by which the substitutions present in M41-R result in attenuation remains undetermined, but it is an important avenue for future research. It is worth noting that proline85 in nsp 10 and valine393 in nsp 14 are conserved among several IBV strains (Fig. 4) as well as in the wider coronavirus family, including SARS-CoV and SARS-CoV-2 (Fig. 9D and E). The residues identified in this study may therefore offer a potential mechanism for the attenuation of other coronaviruses and/or the identification of potential attenuated variants, especially relating to SARS-CoV-2 (80).

The attenuated *in vivo* phenotype exhibited by rIBV M41-R highlights this rIBV as a promising vaccine candidate, further enhanced by the assessment of the stability of the substitutions. *In vitro* and *ex vivo* passaging of both rIBV M41-R isolates demonstrated that all four substitutions were stable up to passage 10. Additionally, the discovery that rIBV M41-R is attenuated *in ovo*, with a hatchability score comparable to that of the mock-infected group, is also very promising. *In ovo* vaccination is advantageous to the poultry industry due to ease of application, ability to control the exact dose delivered, and ability to induce protection from the point of hatching.

## MATERIALS AND METHODS

### Cells and viruses.

Primary CK cells were produced from 2- to 3-week old SPF RIR chickens by Microbiological Services, The Pirbright Institute, Compton (81). All isolates of IBV and rIBVs were propagated in 10-day-old SPF RIR embryonated hens’ eggs. Allantoic fluid was harvested at 24 to 48 hours postinoculation (hpi), clarified by low-speed centrifugation, and quantified by titration on CK cells. IBV isolates Beau-R (38), M41-CK (17), and rIBV BeauR-Rep-M41-Struct (43) have been described previously. All nucleotide positions in the manuscript relate to M41, GenBank accession no. AY851295.1.

### Construction of full-length clone of M41-CK.

The construction of a full-length cDNA copy of the M41-CK genome within a vaccinia virus vector was a multi-step process, as detailed in Fig. 1. The sequence used to generate the full-length clone was an “in-house” sequence generated from M41-CK grown in primary CK cells. The process used previously described reverse genetic techniques which utilize homologous recombination and transient dominant selection to modify the IBV cDNA encoded within a vaccinia virus vector (52, 53). Several plasmids, all based on the pGPTNEB193 vector (53) were required, which were generated by GeneArt (Life Technologies) or through cloning strategies utilizing overlapping PCR mutagenesis. Each stage of the process generated a rVV which acted as a receiver for the next stage; for example, rVV BeauR-Rep-M41-Struct (43) was used as the receiver in a homologous recombination event with a plasmid to delete the Beau-R 5′UTR-nsp 3 sequence. The resulting rVV was then used as the receiver virus in a homologous recombination event with a plasmid to add M41-CK 5′UTR-nsp 2 sequence. Once the full-length cDNA of the M41-CK genome was assembled, rIBV was recovered in CK cells.

### Generation of recombinant IBV containing specific modifications to the nsps.

The methods used to generate rIBV have been described previously (38, 52, 53). Briefly, modified regions of IBV cDNA, specifically with either one or a combination of the following nucleotide modifications, T12137C, C18114G, A19047T, and A20139G, within the pGPT-based plasmids, were introduced into a receiver rVV containing either the M41-R or M41-R-nsp10rep full-length genome sequence by homologous recombination and transient dominant selection. Infectious rIBVs were recovered from an rVV containing the correctly modified sequence in CK cells. Briefly, CK cells were infected with a recombinant fowlpox virus (FPV) expressing T7 RNA polymerase (rFPV-T7) and then transfected with rVV DNA and a plasmid expressing the nucleocapsid gene (pCi-Nuc). The infected/transfected CK cells (P_0_) were incubated until they showed cytopathic effects (CPE). The supernatant containing any potential rIBV was filtered to remove rFPV/T7 and then passaged three more times on CK cells (P_3_). RNA from supernatant harvested from the infected P_3_ CK cells was extracted and analyzed by RT-PCR and spot-sequenced to confirm the presence of rIBV.

### Sequencing library preparation for Hiseq data analysis of M41-CK.

The IBV strain M41-CK was propagated in embryonated hens’ eggs and has a pathogenic *in vivo* phenotype. A volume of 100 ng of total RNA was subjected to library preparation using an NEBNext Ultra mRNA-Seq kit. This was performed without replicates due to restrictions in the amount of starting material available. The resulting library pools were quantified using a NEBNext Illumina library quantitation kit (NEB, Ipswich, MA) before being diluted and loaded onto a single lane of an Illumina HiSeq 4000 (Illumina, San Diego, CA).

### Bioinformatic analysis of Hiseq data.

Trim Galore (http://www.bioinformatics.babraham.ac.uk/projects/trim_galore/) and fastqc (https://www.bioinformatics.babraham.ac.uk/projects/fastqc/) were used to perform initial read QC and quality filtering with a minimum quality score of 30 and minimum read length of 100 bp. Quality cutoffs of 30 and trimming of the first 15 bp and final 45 bp were eliminated from reads before mapping to the virus-specific reference sequence using BWA-MEM (82). Variant calling was performed by two separate methods. First, the SiNPle variant caller (56) was used to identify variants present in the data, using default settings. Alignments of HiSeq reads were corrected for mapping errors and indel correction using LoFreq Viterbi. SNP calling was performed using Lofreq* (57). Sequences were submitted to the NCBI BioSample database, accession number SAMN24687558.

### Ethics statement.

All animal experimental protocols were carried out in strict accordance with UK Home Office guidelines and under license granted for experiments involving regulated procedures on animals protected under the UK Animals (Scientific Procedures) Act of 1986. The experiments were performed in The Pirbright Institute Home Office licensed (X24684464) experimental animal house facilities and approved by the animal welfare and ethical review committee under the terms of reference HO-ERP-01-1. Each experiment used 8-day-old RIR SPF chickens that were hatched and reared in the Poultry Production Unit of The Pirbright Institute, Compton Site. In each experiment the chickens were housed in positive-pressure, HEPA-filtered isolation rooms, and each group was housed in a separate room. Chickens were culled at specific time points by cervical dislocation, a Schedule 1 method.

### Assessment of pathogenicity in chickens.

The first experiment contained groups of chickens infected with rIBVs M41-R-6, M41-R-12, Beau-R, or IBV M41-CK. The second experiment consisted of groups infected with rIBVs M41-R-12, M41R-nsp10rep, M41R-nsp14,15,16rep, or IBV M41-CK. The third experiment consisted of groups infected with rIBVs M41R-nsp10,15rep, M41R-nsp10,14,16rep, M41R-nsp10,15,16rep, M41R-nsp10,14,15rep, M41-K-6, or IBV M41-CK. The fourth experiment consisted of groups infected with rIBV M41R-nsp10,14rep, M41R-nsp10,16rep, M41-K-6, or IBV M41-CK. Every experiment also contained a mock-infected group of chickens as a negative control. Each experiment consisted of groups of 12 chickens inoculated via the conjunctival (eye drop) and intranasal routes with 10^5^ PFU of either rIBV or of IBV in a total of 100 μL serum-free *N*,*N*-Bis(2-hydroxyethyl)-2-aminoethanesulphonic acid (BES) medium or 100 μL serum-free BES for mock infection.

Clinical signs were observed from 1 or 2 dpi to 7 dpi. Snicking was observed by at least two persons over a 2-min period, and the average number of snicks per bird per minute was calculated. Birds were assessed individually for the presence of nasal discharge, watery eyes, wheezing, and rales (a sound emanating from the bronchi), and the percentage of birds positive for rales was calculated. Trachea were removed from three randomly selected birds on 4 and 6 dpi, and from all remaining birds on 7 dpi. From each trachea harvested on 4 and 6 dpi, and on 7 dpi (first experiment only), 10 × 1 mm rings were sectioned (three from the top, four from the middle, and three from the bottom) and the activity of the cilia was observed using a light microscope. The ciliary activity for each trachea was scored using a method previously described by Cook et al. (1999) and Cavanagh et al. (1997) (54, 83).

### Assessment of IBV RNA presence in trachea samples.

One half of the remaining sections of trachea were stored in RNA Later (Ambion) and the other half in phosphate-buffered saline (PBS). The samples stored in RNA Later were used for RNA extraction using an RNeasy Minikit (Qiagen) following the manufacturer’s protocol. A Tissue Lyser II (Qiagen) was used for the homogenization step. RNA was analyzed by either RT-PCR or reverse transcription-quantitative PCR (qRT-PCR). Superscript III/IV and *Taq* DNA polymerase (Invitrogen) were used for RT-PCR, following the manufacturer’s protocol. Random primer (5′-GTTTCCCAGTCACGATCNNNNNNNNNNNNNNN-3′) was used during reverse transcription and IBV-specific oligonucleotides were utilized to amplify the 3′-UTR as previously described (43). The Primerdesign genesig Kit for Avian Infectious Bronchitis Virus (IBV), Advanced Kit was used for qRT-PCR, according to the manufacturer’s protocol. For the latter, 30 μg of tissue was used for the RNA extraction and an on-column DNase treatment (Qiagen) was performed. RNA was quantified using a Nanodrop 1000 and 100 ng RNA was used in each real-time RT-PCR.

### Virus isolation from trachea samples.

Virus reisolation was performed in either *ex vivo* TOCs prepared from 19-day-old SPF RIR embryonated hens’ eggs or in 10-day-old embryonated SPF hens’ eggs. Tracheal rings stored in PBS from each chicken were freeze-thawed and the supernatant clarified by low-speed centrifugation. For isolation in embryonated hens’ eggs, 100 μL of clarified supernatant was used for inoculation and the allantoic fluid harvested at 24 hpi was screened by RT-PCR for the IBV genome. For isolation in *ex vivo* TOCs, tracheal suspensions were prepared by adding 100 μL clarified supernatant to 400 μL TOC medium. TOCs were infected with 150 μL of the tracheal suspension in triplicate. After infection at 37°C for 1 h, an additional 1 mL of medium was added per TOC. TOCs were incubated at 37°C for 6 days, during which they were regularly examined for ciliary activity. The supernatant was harvested from the ciliostatic TOCs, and the remaining supernatant harvested on day 6. Supernatant was screened for viral presence by RT-PCR as previously described.

### Multistep growth curves in CK cells.

Confluent CK cells seeded in 6-well plates were inoculated with either 10^4^ or 10^5^ PFU rIBV/IBV in 0.5 mL BES medium and incubated for 1 h at 37°C in 5% CO_2_. Following attachment, cells were washed twice with PBS to remove residual virus, after which 3 mL serum-free BES medium was added per well. Extracellular virus was harvested at 1, 24, 48, 72, and 96 hpi, and assayed by titration in CK cells.

### Multistep growth curves in *ex vivo* TOCs.

Tracheal organ cultures were prepared from 2- to 3-week old SPF RIR chickens as previously described (84). In triplicate, each TOC was inoculated with 10^4^ PFU of either rIBV or IBV in 0.1 mL TOC medium and incubated for 1 h at 37°C. The inoculum was removed and TOCs were washed twice with PBS to remove residual virus, after which 1 mL TOC medium was added. Infected TOCs were incubated at 37°C with 7 to 8 revolutions per h. Extracellular virus was harvested at 1, 24, 48, 72, and 96 hpi, and assayed by titration in CK cells.

### Assessment of ciliary activity in *ex vivo* TOCs.

TOCs were prepared from 19-day-old SPF RIR embryonated hens’ eggs as previously described (84). Each TOC was inoculated with 5 × 10^4^ PFU in 500 μL of TOC medium of rIBV or IBV in replicates of 11, and incubated for 1 h at 37°C. After incubation an additional 500 μL of TOC medium was added per TOC, followed by incubation at 37°C with 7 to 8 revolutions per h. The ciliary activity of each TOC was assessed at regular intervals and scored according to methods described by Cook et al. (1999) and Cavanagh et al. (1997) (54, 83).

### Serial passaging in CK cells.

Confluent CK cells in 6-well plates were washed with PBS and inoculated in triplicate with rIBV or IBV diluted 1/10 in 500 μL BES medium, or 500 μL BES medium for mock infection, and incubated for 1 h at 37°C in 5% CO_2_. Following attachment, cells were washed twice with PBS to remove residual virus, after which 3 mL serum-free BES medium was added per well. Extracellular virus was harvested 24 hpi and used for the following passage using the same procedure described above. Passage 9 virus was amplified in T75s to generate a larger working stock of the passage 10 virus, using the same protocol except for an additional 24 h of incubation at 37°C. At passage 5 and 10 the presence of rIBV in the supernatant was confirmed by RT-PCR using IBV-specific oligonucleotides targeting the 3′ UTR (43). The passage 10 viruses were spot-sequenced to assess the stability of the following nonsynonymous substitutions: nsp 10 (C12137U), nsp 14 (G18114C), nsp 15 (U19047A), and nsp 16 (G20139A).

### Serial passaging in TOCs.

TOCs were prepared from 19-day-old SPF RIR embryonated hens’ eggs as previously described (84). Three TOCs were pooled in a single tube and checked for vitality before use. TOCs were washed with PBS before being inoculated with 10^5^ or 10^6^ PFU/mL for each rIBV in a volume of 200 μL in 4 replicates, and tubes were incubated standing for 1 h at 37°C. After incubation, TOCs were washed with PBS to remove residual virus and 1 mL of TOC medium was added per tube. Supernatants were harvested 24 hpi and used for direct inoculation of the next passage following the same procedure described above. At passage 10 supernatants were tested for rIBV by titration in CK cells and by RT-PCR targeting the 3′ UTR (43). Viruses were spot-sequenced at passage 10 to assess the stability of the nonsynonymous substitutions: nsp 10 (C12137U), nsp 14 (G18114C), nsp 15 (U19047A), and nsp 16 (G20139A).

### Hatchability and safety trial *in ovo*.

Groups of 33 eggs each were incubated under standard conditions. After 18 days of incubation, the eggs were taken out of the incubator and inoculated with 10^1^ or 10^4^ EID_50_ rIBV in 0.1 mL PBS. After inoculation the eggs were incubated under standard conditions and allowed to hatch. The number of live chickens at 1-day post-hatch per group was determined.

### Protein modeling.

Predicted structures of the M41-CK nsps and the M41-R-like nsps containing the substitutions C12137U (Pro85Leu) in nsp 10, G18114C (Val393Leu) in nsp 14, U19047A (Leu183Ile) in nsp15, and G20139A (Val209Ile) in nsp 16 were generated using SWISS-MODEL (85). Existing cryo-EM and crystal structures on RCSB Protein Data Bank (https://www.rcsb.org/) were used as the templates. RCSB PDB: 2XYV was used as the backbone for the nsp 10 and nsp 16 models. The structural model of nsp 14 used RCSB PDB: 5C8T as a template. The PDB structures were then exported and rendered using PyMol (https://pymol.org/2/) (86). Rendered structures were also overlaid and point mutations isolated and enhanced.

### Statistics.

All statistical analyses were performed using GraphPad Prism version 8.0. Normality and the standard deviation of each data set were assessed prior to each statistical test.
